# Results from the Strong Families Start at Home/Familias Fuertes Comienzan en Casa: feasibility randomised control trial to improve the diet quality of low-income, predominantly Hispanic/Latinx children

**DOI:** 10.1017/S1368980023000174

**Published:** 2023-04

**Authors:** Alison Tovar, Katelyn Fox, Kim M Gans, Patricia Markham Risica, George D Papandonatos, Andrea Ramirez, Amy A Gorin, Tayla von Ash, Ernestine Jennings, Kelly Bouchard, Karen McCurdy

**Affiliations:** 1 Department of Behavioral and Social Sciences, Brown School of Public Health, Box G-121S Rm 813, Providence, RI 02912, USA; 2 Department of Nutrition and Food Sciences, University of Rhode Island, Kingston, RI, USA; 3 Department of Human Development and Family Sciences, University of Connecticut, Storrs, CT, USA; 4 Department of Biostatistics, Brown University, Providence, RI, USA; 5 Department of Psychological Sciences, University of Connecticut, Storrs, CT, USA; 6 Department of Psychiatry and Human Behavior, The Warren Alpert Medical School, Brown University, Providence, RI, USA; 7 Department of Human Development and Family Science, University of Rhode Island, Kingston, RI, USA

**Keywords:** Diet quality, Children, Feeding, Food parenting practices, Home food environment, Hispanic/Latinx

## Abstract

**Objective:**

To describe the feasibility, acceptability and results of Strong Families Start at Home, a 6-month pilot trial of a home-based food parenting/nutrition intervention.

**Design:**

Pilot randomised controlled trial.

**Setting:**

Participants received six visits with a community health worker trained in motivational interviewing (three home visits, three phone calls); an in-home cooking or reading activity; personalised feedback on a recorded family meal or reading activity; text messages and tailored printed materials.

**Participants:**

Parents and their 2–5-year-old child were randomised into intervention (responsive food parenting practices/nutrition) or control (reading readiness) groups.

**Results:**

Parents (*n* 63) were mostly mothers (90 %), Hispanic/Latinx (87 %), born outside the USA (62 %), with household incomes <$25 k (54 %). Despite delivery during COVID-19, 63 % of dyads were retained at 6 months. The intervention was delivered with high fidelity. All parents in the intervention group (*n* 24) expressed high levels of satisfaction with the intervention, which produced positive treatment effects for whole and total fruit component Healthy Eating Index-2015 scores (point estimate (PE) = 2·14, 95 % CI (0·17, 1·48); PE = 1·71, 95 % CI (0·16, 1·47), respectively) and negative treatment effects for sodium (PE = -2·09, 95 % CI (−1·35, −0·04)). Positive treatment effects also resulted for the following food parenting practices: regular timing of meals and snacks (PE = 1·08, 95 % CI (0·61, 2·00)), reducing distractions during mealtimes (PE = -0·79, 95 % CI (−1·52, −0·19)), using food as a reward (PE = -0·54, 95 % CI (−1·35, −0·04)) and providing a supportive meal environment (PE = 0·73, 95 % CI (0·18, 1·51)).

**Conclusion:**

Given the continued disparities in diet quality among low-income and diverse families, continued efforts to improve child diet quality in fully powered intervention trials are needed.

The diet quality of US children is poor, with few fruits and vegetables (F&V) and whole grains, and overconsumption of energy-dense snacks and beverages, especially among low-income and ethnically diverse families^(1)^. This dietary pattern is associated with significant increases in markers of cardiometabolic risk in young children^(2)^, highlighting the need for effective interventions.

The preschool years are a critical time for shaping food preferences, which track into adulthood^(3)^. Within the home environment, parents provide access and availability to foods and interact with preschool children in ways that can promote the development of eating habits^(4)^. Responsive food parenting practices, like involving children in meal planning/preparation, increases the likelihood they will try the foods being prepared. Providing a positive structure (such as having healthy foods available in the home) and modelling intake of healthy foods have been associated with increases in children’s intake of healthy foods^(5)^. In contrast, coercive food parenting practices like pressuring a child to eat, using a favoured food as a threat or bribe, undermines a child’s ability to self-regulate food intake and may hinder the development of a healthy diet and contribute to excess energy intake^(6)^. A 2021 expert panel report highlighted the importance of autonomy, structure and repetition to help young children develop healthy eating habits^(4)^.

In addition, not all children respond to food environments in the same way and certain appetitive traits, such as satiety responsiveness (sensitivity to internal satiety signals), food responsiveness (sensitivity to external food cues) and food fussiness may help explain some of these differences^(7)^. Children with overweight/obesity often have lower satiety responsiveness, higher food responsiveness and higher enjoyment of food than children with a healthy weight^(8)^. A 2021 meta-analysis suggested that appetitive traits may provide a novel intervention target, with potential implications for clinical practice and population health^(9)^. Furthermore, child–parent food interactions are bidirectional in which child behaviours can influence certain food parenting practices and vice versa^(10,11)^. Tailoring interventions on child appetitive traits can help parents have realistic expectations of their child’s behaviour, which can help reduce the stress related to feeding children, prioritise certain practices, incorporate certain foods more easily and help parents decide which recommendations to focus on for goal setting.

Given the important influence of appetitive traits and food parenting practices on children’s diet, the development of effective interventions that include these factors is essential. To date, interventions focused on food parenting practices have not always utilised parenting theories to inform the intervention^(12)^ and typically address what parents should not be doing, contributing to parents feeling judged, while not encouraging what they should be doing. Furthermore, previous interventions have had limited success in reaching busy, low-income, ethnically diverse parents, keeping them engaged and changing their child’s diet^(13,14)^. Although there have been some home-based interventions aimed at the prevention or treatment of overweight/obesity that included components on healthy eating, few have focused on improving diet quality of diverse families. A 2021 review identified fourteen family-based, obesity prevention interventions (2015–2021) tailored for 2–5-year-old children of racial and ethnic minoritised populations. Of the identified interventions, eight reported diet-related outcomes, all of which found improvements^(15)^. However, most studies that analysed diet did not assess overall diet quality, and instead focused on intake of specific food groups or energetic intake. Thus, there is a need for home-based interventions to incorporate comprehensive nutrition alongside food parenting strategies to assess impact on overall diet quality.

This paper describes the results of *Strong Families Start at Home/Familias Fuertes Comienzan en Casa*, a home-based pilot intervention with low-income, predominantly Hispanic/Latinx families that aims to empower parents to identify and implement responsive food parenting practices, tailor their practices to their child’s appetitive traits and utilise healthy food shopping and preparation strategies. The primary objectives of this study were to determine the feasibility of the study protocols, recruitment, the acceptability and fidelity of the intervention and its preliminary efficacy on changes in children’s diet quality and food parenting practices compared to an attention control group.

## Methods

This 6-month pilot randomised controlled trial (July 2019 – March 2021) randomly assigned parent–child dyads, using block randomisation stratified by ethnicity, into one of two groups: the intervention group that focused on responsive food parenting practices/nutrition and the attention control group, which focused on reading readiness. A community advisory board advised on all aspects of the study including design, recruitment and intervention implementation. The study was approved by the Institutional Review Board of the University of Rhode Island Institutional Review Board (HU1819-007). The protocol paper with details on the study design, measures, training protocols, sample size and intervention has been published elsewhere, but a brief description is provided below^(16)^.

### Recruitment and eligibility

Participants were recruited through a variety of active and passive strategies. For active recruitment, Special Supplemental Nutrition Program for Women, Infants and Children (WIC) nutritionists collected the contact information of interested participants, which was then passed to research staff for follow-up. Study staff members also recruited parents in WIC waiting rooms. Passive recruitment strategies included placing flyers and sign-up sheets in childcare settings and doctors’ offices. To be eligible, the participant had to be the primary caregiver of a child between 2 and 5 years of age, ≥18 years old, speak English or Spanish and have a phone that could video record. Caregivers were ineligible if their child had a medical condition impacting food intake. At baseline, parents provided informed consent, completed twenty-four hour recalls and surveys.

### Intervention

The intervention consisted of 3 monthly home visits (60–75 min) followed by 3 monthly phone calls (30–45 min), conducted by trained bilingual community health workers (CHW). At each visit, the CHW used motivational interviewing (MI) to elicit and reinforce any language indicating the parent’s desire, ability, reason, need or commitment to change food parenting practices. MI was incorporated using open-ended questions, affirmations, reflections and summaries to actively involve the parent in the conversation. The CHW prompted the participant to choose a goal that they thought would be helpful with regard to their child’s eating, based on the information reviewed during the session. Collaboratively, the CHW and parent developed a food parenting and nutrition plan that included specific goal(s) aimed at food parenting practices (defined for participants as ‘how they interact with children around meals’), and the home food environment, reasons for the plan, potential barriers to completing the plan and some possible solutions (including social supports). Each visit was accompanied by written materials which included a 2–4-page handout highlighting nutrition and food parenting guidance, such as creating family routines around healthy eating, empowering children to make healthy choices, choosing and preparing healthy family meals and snacks on a budget, meal planning and tips on how to involve children in family meal planning and preparation. Parents also received a tailored handout if their child was categorised as low or high on satiety responsiveness (sensitivity to internal satiety signals), food responsiveness (sensitivity to external food cues) and food fussiness, based on the child’s appetitive traits collected using the Child Eating Behavior Questionnaire at baseline^(17)^. For this study, we use 3 subscales: food responsiveness (sensitivity to external food cues), satiety responsiveness (sensitivity to internal satiety signals) and food fussiness, since they have consistent associations with either BMI or diet quality^(18)^. Children were categorised as high in each category based on the following scores: food responsiveness >2·8, satiety responsiveness >2·8 and food fussiness >3·0^(19,20)^. For example, parents of children who were identified as ‘food fussy’ received tailored information on responsive strategies such as limiting pressure to eat, offering guided choice, increasing child involvement in food preparation and using repeated exposure to new foods. If the child did not fall into any of these categories, they did not receive the additional information. Text messages were sent two times/week (Mosio, Inc), during the 6-month intervention with messages relating to the objectives targeted during that month’s visit.

During the first and third home visit, the CHW discussed one of the family’s video-recorded meals, as described below, and during the second visit, the CHW, together with a culinary school intern, led a hands-on food preparation of a recipe selected during the first home visit.

### Video-recorded meals

Participants were asked to video record a family meal and send the video to the research team via Google Drive or WhatsApp. WhatsApp uses end-to-end encryption, which ensures that only the people in the chat can read or listen to what is sent. Google Drive Data are encrypted in-transit and at-rest. Videos were downloaded to a secure password-protected server. Trained nutrition students coded the video using a tool developed for this project. Coders tallied their observations of eleven food parenting practices, with operational definitions based on Vaughn and Haycraft^(21)^. This tool was previously reviewed by six experts in the field of child food parenting and, using 10 videos, demonstrated preliminary validity and reliability (re-test reliability = ICC 0·93, English video’s inter-rater reliability = ICC 0·82 and Spanish video’s inter-rater reliability = ICC 0·93) in identifying food parenting practices of video-recorded family meals. The coders identified video segments that were representative of responsive and non-responsive food parenting practices and provided those clips to the CHW along with a feedback sheet that included information as to why that practice was responsive (to reinforce the practice) or non-responsive (to reflect on the practice). During the first and third home visits, the CHW and parent watched the segments of the video-recorded family meal previously identified by the researcher. Using MI, the CHW elicited the parent’s thoughts and beliefs regarding practices used during the meal video and facilitated the development of a plan to reinforce or improve the practices.

### Attention control group

The comparison group received an attention-matched intervention about school readiness promotion adapted from R.E.A.D.Y. (Read Educate and Develop Youth) designed by the Michigan Department of Education. Parents received the same intervention components as the intervention group, pertinent to school readiness instead of nutrition. This included video assessment of a parent reading or completing an activity with their child, and three-monthly phone calls to check in on progress related to their goals. Parents also received text messages as well as printed materials.

### Intervention changes due to COVID-19

Eight months into the study intervention period, the COVID-19 pandemic and associated closures began. After research was allowed to resume and in consultation with the National Institutes of Health Program Officer, several changes were implemented to continue the intervention under socially distanced circumstances. All home visits became virtual using FaceTime or WhatsApp, depending on participant preference. In place of the in-home cooking session, we developed boxes with the recipe ingredients, a cookbook and other materials (cups and child apron) and delivered them to participants’ homes. Shortly after dropping off the food box, we sent a newly created cooking video tutorial that included the steps to make the recipe, as well as a demonstration on how to involve children in the recipe preparation. After watching the video, we asked the participants to make the recipe with their child before the next virtual visit. During the following virtual visit, the CHW checked in with the participant about their cooking experience. Of 139 home visits conducted, 47 % were conducted virtually due to COVID-19. We also added a new handout for participants that included tips about COVID-19 related to grocery shopping, maintaining structure at home, activities for children and information on local food assistance programs.

### Measures and data collection

Participants had two 90-min home measurement visits (baseline and 6-month follow-up). At both visits, trained study staff administered a survey that included food parenting questions, home food inventory and other health behaviour assessments. They also measured the enrolled parent and child height and weight and completed one of two 24-h dietary recalls as described below. Upon completion of the baseline visit, parents received a $35 gift card and study staff scheduled a second dietary recall to be completed over the phone. After completing the second recall, participants were compensated with a $15 gift card and randomised. At follow-up, participants received a $50 gift card for the measurement visit and $35 for completing the second recall. Data collectors were blinded to experimental condition. After COVID-19, all data collection procedures were conducted virtually over the phone.

#### Feasibility

Participant retention and intervention fidelity were used as the feasibility measures. Retention was measured through CONSORT participant tracking logs which included the number of visits scheduled and completed by the participant as well as documentation of the reason for study discontinuation. The study was considered feasible if 80 % of the participants were retained in the intervention at 6 months. Intervention fidelity was measured using audio recordings of each visit, CHW self-reported fidelity checklists for home visits and phone calls, goal setting and planning documentation, participant satisfaction surveys, completion of the video recording of a family meal and qualitative interviews with participants. Fidelity was assessed across the 5 domains recommended in the National Institutes of Health Behavior Change Consortium treatment fidelity framework to ensure intervention fidelity across: (1) Study design; (2) Training; (3) Intervention delivery; (4) Intervention receipt and (5) Intervention enactment^(22)^ (Table 1).

**Table 1:** Intervention fidelity assessment by national institutes of health (NIH) behavior change consortium (BCC) treatment fidelity framework domain

Monitoring tool	NIH-BCC domain				Intervention enactment
	Study design	Training	Intervention delivery	Intervention receipt	
Participant tracking log	x				
Weekly intervention team meetings	x	x	x	x	x
CHW self-reported fidelity checklist	x	x	x	x	
Goal setting and planning documentation			x	x	
Satisfaction survey		x	x		x
Audio-recording					
Fidelity checklist		x	x		x
MITI		x	x		
Completion of video recording				x	x
Post-intervention interviews				x	x

#### Fidelity to study design

The dose of intervention received by participants in the intervention and control groups was measured. To measure dose, the CHW self-reported fidelity checklist was used, which included questions on participant attendance, the length of time for each visit and whether participants received the text messages and printed materials.

#### Fidelity to training

Each CHW was required to complete 16 h of standardised training in which they demonstrated ability to deliver the scripted intervention and adhere to the core principles of MI. CHW also demonstrated ability to follow the quality assurance protocol including entering self-reported fidelity and satisfaction checklists in REDCap and uploading audio recordings. CHW skills were assessed and reinforced overtime through quality assurance monitoring, weekly intervention team meetings and periodic individual supervision.

#### Fidelity to intervention delivery

Fidelity checklists were reviewed on a weekly basis. Any deviations from protocols were discussed on an individual basis and during weekly meetings. In addition to CHW and participant report, fidelity of intervention delivery was also assessed by reviewing audio recordings of the visits. To measure adherence to the script and protocols and assess the quality of communication, the project coordinator randomly assigned 10 % of audio recordings to a research assistant who was trained to assess and document fidelity using a checklist created for each visit. The objective fidelity checklists assessed whether the CHW discussed key concepts, conducted the main activity (video review or cooking session), solicited participants thoughts on, and understanding of, the information provided, and completed the goal-setting form. Research assistants also provided subjective feedback on the overall impression and tone of the visit. The Motivational Interviewing Treatment Integrity Code (MITI 3·1·1) was used to determine CHW MI adherence. A trained rater randomly coded 10 % of the sessions, selecting 20-min segments of the recorded sessions using the MITI 3·1·1. A second trained rater double coded a selection of these sessions.

#### Fidelity to intervention receipt

Participants’ intervention receipt and ability to comprehend the information provided and use skills taught in the intervention was measured using the CHW self-reported fidelity, goal setting and planning form. CHW documented whether participants demonstrated ability to prepare a meal with their child, set a goal related to the intervention content and identify barriers and facilitators to achieving the goal. Participants reported whether they read the text messages or written materials and rated their confidence in their ability to achieve their goals. CHW also rated how engaged participants were in the session and documented any factors that may have influenced their ability to comprehend or use the information provided.

#### Fidelity to intervention enactment

Data on whether participants used the information and skills taught in the intervention in relevant day-to-day settings was assessed. Participants reported the likeliness to make changes based on each activity as part of their satisfaction survey. Participant reports of a change in attitudes, knowledge and skills and/or behaviours were also coded as part of the audio recorded visits and follow-up interview.

#### Acceptability

For acceptability, participants completed a short satisfaction survey after each intervention session that asked about satisfaction with the intervention components and sessions including the time duration, materials, activities and feedback received. In the final follow-up survey, they were also asked about satisfaction with the overall intervention and each intervention component. Post-intervention, semi-structured interviews were conducted with twenty-two participants to understand what parents liked and did not like about the intervention.

#### Primary outcome

Child diet quality was assessed using two 24-h dietary recalls. The recommended multiple-pass approach was used to provide various opportunities for the participant to recall food intake. Study staff provided the bilingual Food Measurement Aids for Infants and Toddlers booklet during the assessment visits to help participants report portions or volume of foods and beverages consumed more accurately. The recalls were collected and analysed using the Nutrition Data System for Research software. Efforts were made to conduct dietary recalls on a weekday and a weekend day to reflect changes in dietary patterns. Healthy Eating Index-2015 (HEI) total and component scores were derived using the National Cancer Institute simple HEI scoring algorithm method. Total HEI score ranges from 0 to 100 with higher scores reflecting higher diet quality and a score of 80 or higher reflecting a high-quality diet among preschool-aged children^(23)^. Scoring reflects intake per 1000 calories, except for saturated fats and added sugars which are a percentage of energy. Higher scores for adequacy components (total fruits, whole fruits, total vegetables, greens and beans, whole grains, dairy, total protein foods, seafood and plant protein components) reflect higher intake, whereas higher scores for moderation components (refined grains, sodium, added sugar and saturated fat components) reflect lower intake.

#### Secondary outcomes

Three measures were used to capture food parenting practices. First, the Food Parenting Inventory, a fifty-three-item questionnaire, which has shown good reliability and validity among Hispanic/Latinx parents^(24)^. Second, one subscale from the Comprehensive Feeding Practices Questionnaire^(25)^, Healthy Eating Guidance, was used. Items are rated on a five-point Likert scale ranging from 1 (*never*) to 5 (*always*) and 1 (*disagree*) to 5 (*agree*). Higher subscale scores indicate greater use of that food parenting practice. The third measure, Parent Socioemotional Context of Feeding Questionnaire, captured the socio-emotional context of meals^(26)^. This twenty-four-item measure has been validated with mothers of 4–8-year-old children and measures the extent to which a feeding environment is autonomy-supportive, structured, controlling or chaotic, and has demonstrated good internal consistency and construct validity. Parents rated items regarding their behaviours and emotions at mealtimes on a five-point Likert scale ranging from 1 (extremely untrue) to 5 (extremely true). Scores were averaged for each subscale, with higher scores indicating greater endorsement of each construct.

### Analysis

#### Feasibility and acceptability

For all feasibility and acceptability measures, descriptive statistics were used to summarise the data. A rapid qualitative analysis^(27)^ was conducted to summarise the interview data. During each interview, thorough notes were taken and immediately after the interview, the data were summarised into post-interview notes and added to a template based on the interview guide along with major themes that emerged during the interview. These themes were then discussed with the larger team. Once all interviews were completed, the summary data from each of the interviews were integrated into one document and the final themes were identified.

#### Primary and secondary outcomes

Between-group comparisons of key socio-demographic characteristics as well as of primary and secondary outcomes (Table 2) were used to assess randomisation imbalances at baseline. Potential moderators considered were parental age, marital status, income, race/ethnicity, birth country, years in the USA, household composition, household chaos as well as child age, gender, BMI, and childcare attendance. The collection of these data is described in the protocol paper^(16)^.

**Table 2:** Strong Families Start at Home: baseline participant demographic characteristics, overall and by intervention arm

Characteristic	Overall (*n* 63)		Control group (*n* 30)		Intervention group (*n* 33)		*P* value
	*n*	%	*n*	%	*n*	%	
Gender of target child (Female)	28	44·4	10	33·3	18	54·5	0·15
Gender of parent (Female)	58	92·1	28	93·3	30	90·9	1·00
Relationship to child							1·00
Mother	57	90·5	27	90·0	30	90·9	
Father or other	6	9·5	3	10·0	3	9·1	
	Mean	sd	Mean	sd	Mean	sd	
Parent age	34·48	7·59	34·27	8·36	34·67	6·94	0·84
	*n*	%	*n*	%	*n*	%	
Ethnicity (Hispanic/Latinx)	55	87·3	26	86·7	29	87·9	1·00
Race							0·89
White	24	38·1	12	40·0	12	36·4	
Multiracial	11	17·5	5	16·7	6	18·2	
Unknown	15	23·8	6	20·0	9	27·3	
Other*	13	20·6	7	23·3	6	18·2	
Country of birth							0·58
United States	24	38·1	13	43·3	11	33·3	
Other	39	61·9	17	56·7	22	66·7	
Language used at home							0·39
English	21	33·3	12	40·0	9	27·3	
Spanish	41	65·1	18	60·0	23	69·7	
Missing	1	1·6	0	0·0	1	3·0	
Annual household income							0·22
Less than $25 000	34	54·0	20	66·7	14	42·4	
Between $25 000–74 999	20	31·7	6	20·0	14	42·4	
More than $75 000	3	4·8	1	3·3	2	6·1	
Unknown	6	9·5	3	10·0	3	9·1	
Highest level of education							0·66
Less than eighth grade	9	14·3	5	16·7	4	12·1	
High school†	23	36·5	12	40·0	11	33·3	
College‡	31	49·2	13	43·3	18	54·5	
Employment status							0·27
Employment full time	15	23·8	5	16·7	10	30·3	
Employment part time	13	20·6	7	23·3	6	18·2	
Other§	35	55·6	18	60·0	17	51·5	
Marital status							0·27
Married	28	44·4	13	43·3	15	45·5	
Not married‖	25	39·7	10	33·3	15	45·5	
Missing	10	15·9	7	23·3	3	9·1	
Currently living with a spouse/partner							0·79
Yes	43	68·3	20	66·7	23	69·7	
No	17	27·0	9	30·0	8	24·2	
Missing	3	4·8	1	3·3	2	6·1	
Number of other adults living in home							0·65
None	5	7·9	3	10·0	2	6·1	
One	15	23·8	6	20·0	9	27·3	
Two or more	42	66·7	21	70·0	21	63·6	
Missing	1	1·6	0	0·0	1	3·0	
Number of children (<18 years) living at home							0·42
One	19	30·7	11	36·7	8	24·2	
Two or more	44	69·8	19	63·3	25	75·8	
Currently pregnant							0·41
Yes	7	11·1	5	16·7	2	6·1	
No	49	77·8	22	73·3	27	81·8	
Missing	7	11·1	3	10·0	4	12·1	
Food assistance							0·57
Yes¶	51	81·0	24	80·0	27	81·8	
No	11	17·5	5	16·7	6	18·2	
Missing	1	1·6	1	3·3	0	0·0	
Food insecurity							0·11
Yes**	28	44·4	17	56·7	11	33·3	
Target child attends childcare							0·99
Yes	20	31·7	9	30·0	11	33·3	

* Includes: Black/African American, American Indian/Alaskan Native, Asian, Hawaiian/Other Pacific Islander, other.

† Includes some high school, high school graduate or General Educational Diploma (GED), post high school trade or technical school.

‡ Includes some college and college graduate or higher.

§ Other includes employed seasonally, unemployed/looking for work, student, homemaker and people with disabilities.

‖ Not married includes never married, separated, divorced and widowed.

¶ Includes the Supplemental Nutrition Assistance Program (SNAP), Supplemental Nutrition Program for Women, Infants and Children (WIC), free/reduced price school meal, soup kitchen, food pantry.

** Includes any level of food insecurity (‘We worried whether our food would run out before we got money to buy more; the food we bought just didn’t last, and we didn’t have money to get more.’).

Inverse probability of retention weighting (IPRW) was used to correct observed outcomes for participants’ differential propensity to drop out. We estimated the response rate in each arm conditionally on baseline characteristics in Table 2, divided these propensity scores by the overall retention rates in each arm and inverted the resulting ratios to create stabilised weights of unit mean under the assumption of no model misspecification. We assessed the success of the IPRW approach in reducing selection bias by comparing standardised mean differences (SMD) in baseline characteristics between respondents and non-respondents. A SMD of 0·2 pooled standard deviations after weighting was used as an indication that the propensity-weighting procedure was successful in balancing an individual characteristic across those retained and those that dropped out. The stabilised weights were then used to estimate normal linear regression models for between-arm differences and the outcomes of interest via the survey package in R 4·0·2. Both primary and secondary outcome analyses controlled for baseline values of the corresponding outcome via covariate adjustment. In addition, any variables that showed persistent imbalances even after propensity weighting were added to each outcome model to help control for any residual bias due to selective attrition. Familywise error rates were controlled at *α* = 0·05 for primary and secondary outcomes separately using Holm’s sequentially rejective procedure.

## Results

Participants (*n* 63) were mostly mothers (90 %), Hispanic/Latinx (87 %), born outside the USA (62 %), with household incomes <$25 k (54 %), and employed full or part-time (45 %) with 33 % homemakers and 11 % unemployed. Approximately half reported being married (44 %) with 27 % reporting they did not live with a spouse/partner and 32 % had a child in childcare. At baseline, 44 % reported some level of food insecurity and 84 % participated in federal food assistance programs (e.g. SNAP, WIC, Free or Reduced-Price School Meals). (Table 2) At baseline, no significant differences between intervention and control groups were detected for any of the socio-demographic characteristics (Table 2). In addition, there were no significant differences in children’s diet quality (total and component HEI-2105 scores) by intervention arm at baseline.

**Fig. 1:**
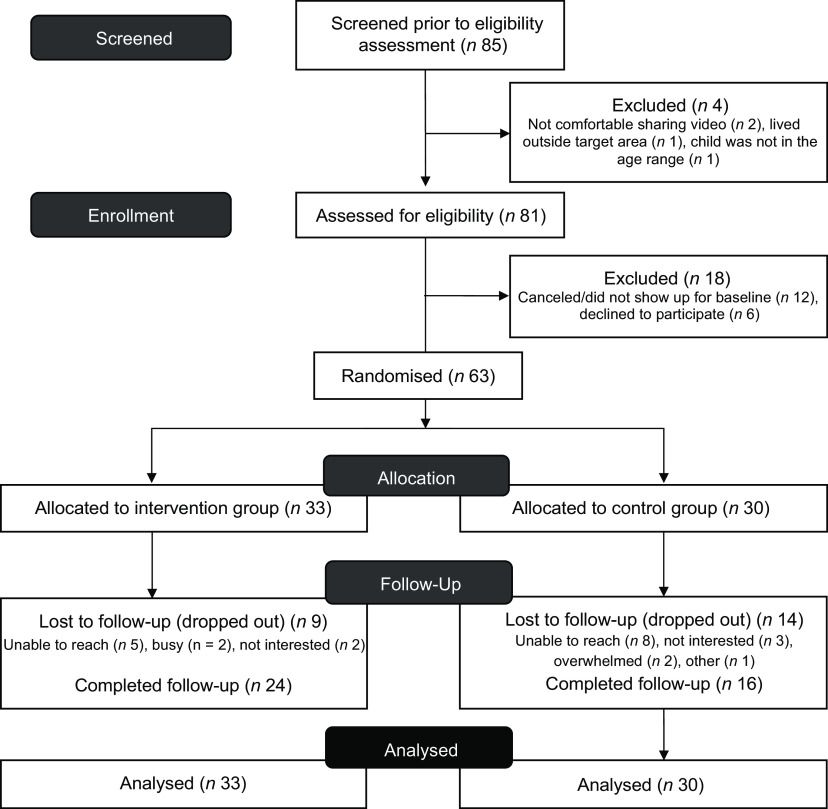
Consort flow diagram of the Strong Families Start at Home Study

The goal was to recruit a total of sixty parents to achieve a final sample of 50, assuming a 20 % attrition. Eighty-five participants of which twenty two were either not eligible or interested were recruited and sixty-three participants were randomised. Of these, 63 % of the participants were retained at study completion, as shown in the CONSORT diagram (Fig. 1).

### Feasibility

#### Fidelity to the study design

With regard to dose delivered, CHW conducted a total of 252 sessions with the participants (153 in the responsive food parenting/nutrition group and ninety-nine sessions in the reading readiness group) with a completion rate of 97·9 %. With regards to dose received, participants received an average of 4·5 ± 2·1 visits (home/video or in-person) with an average session length time of 48·8 min. Participants in the responsive food parenting/nutrition intervention reported high rates of receiving between-visit text messages (95 %) and mailed handouts (87 %).

#### Fidelity to training

CHW demonstrated maintenance of knowledge and skills throughout the intervention. Weekly meetings allowed for the CHW to discuss their experiences with delivering the intervention, major challenges and other anecdotal successes with the rest of the team.

#### Fidelity to intervention delivery

CHW reported fidelity to intervention delivery was high. MI was used across 91 % of all visits, and goal-setting sheets were completed by CHW 89 % of the time. Research assistants coded fifty session audio recordings to objectively assess fidelity. Adherence to protocols was high during home visits (88 % adherent). CHW reviewed the intended material, followed the instructions for introduction and building rapport and completed activities. For phone calls, the CHW adherence was acceptable, although lower at 64 %. Specific components that had low adherence (<50 %) during phone call visits included CHW introducing themselves and/or reminding the participant of who they are and asking participants if they had questions about the materials.

#### Fidelity to intervention receipt

Overall, participants reported recieiving and engaging with the information. Participants in the responsive food parenting/nutrition intervention reported high rates of reading between-visit text messages (95 %) and reading mailed handouts (73 %). CHW rated participants engagement an average of 9·7 on a scale of 1–10 with 10 being the most engaged.

#### Fidelity to intervention enactment

Most participants (86 %) said it was highly likely they would make changes based on the food parenting practices video feedback from home visit 1, but fewer (55 %) reported this after home visit 3. It is important to highlight that not all participants in the responsive food parenting practices/nutrition group who completed home visit 3 sent the video recording (24 *v*. 15 participants), therefore, some of them did not receive individualised feedback based on the video.

### Acceptability

Participants in the responsive food parenting/nutrition group reported that they were satisfied with the different components after each visit. For example, they expressed high levels of satisfaction (‘satisfied’ or ‘very satisfied’) with the video feedback (home visit 1 and home visit 3 = 100 %). Using a 1–4 scale, 4 being very useful, participants in the group found the materials (score: 3·6), the coach (score: 3·5), the cooking activity (score: 3·6), the video feedback (score: 3·4) and the text messages (score: 3·5) useful. All participants from the group who completed the satisfaction survey after home visit 2 (*n* 22), expressed satisfaction with the home visit, rated 3·8, and the cooking activity, rated 3·9, on a 1–4 scale, 4 being very useful. Participants in the reading readiness group were also very satisfied with their intervention (score: 3·9) and found the materials (score: 3·9), the coach (score: 3·7), the video feedback (score: 3·9) and the text messages (score: 3·9) useful.

After follow-up assesments were complete, semi-structured interviews were conducted with twenty-two of the twenty-four participants in the responsive food parenting/nutrition group that completed the study. All interviewed participants stated that they were very satisfied with the intervention and that they would participate again. Activities that the parents described enjoying the most included: (1) home-cooking sessions (for those that were able to have it in their homes), receiving the food box and watching the video on how to prepare the meal with their child (for those who completed during COVID-19) as this allowed them to connect and include their child in the kitchen; (2) working one-on-one with a CHW: parents enjoyed having conversations with their ‘coaches’; they felt heard and were able to set important goals that they worked towards; (3) video-based feedback: they shared that it was ‘eye-opening’ to see themselves and to learn about areas that they are doing well on and areas they can further improve; (4) text messages: they liked the quantity and nature (encouraging) of the messages, the ideas on how to involve kids and the recipes and (5) written materials: parents posted handouts on the fridge, shared them with their families and found them very informative.

### Inverse probability of retention weighting

Higher retention rates were associated with several baseline characteristics, i.e. born outside the USA, receiving the intervention in Spanish, Spanish language use at home, at least some college-level education, no receipt of any food assistance, no childcare attendance, higher child involvement in food preparation and higher HEI-2015 fatty acids and saturated fat components (all *P* < 0·10) (Additional Files 1 and 2). Three of the baseline variables (HEI-2015 saturated fat, child involvement in food preparation and receipt of any food assistance) showed persistent imbalances even after propensity weighting and were added to all survey-weighted linear regression models examining outcome differences by study arm to help control for residual bias due to selective attrition.

### Primary outcome: diet quality

The mean HEI-2015 score for participating children was 61·06 (sd: 12·04) at baseline (Table 3). Treatment effects for primary outcomes are shown in Table 4. Although this was a pilot feasibility trial, not powered to detect statistically significant differences, for total and whole fruit the effect sizes were significantly different from zero, indicating increases in fruit consumption (point estimate (PE) = 1·71, SE = 0·67, 95 % CI (0·16, 1·47)), PE = 2·14, se = 0·83, 95 % CI (0·17, 1·48), respectively), and for sodium (PE = -2·09, se = 0·97, 95 % CI (−1·35, 0·04)), indicating an increase in sodium consumption for participants who were in the food parenting/nutrition *v.* those in the reading readiness group. Although they were all in the ‘large’ range according to Cohen’s nomenclature (*δ* = 0·70–0·83), none survived Holm’s multiplicity adjustment (Table 4). Whole grains changed in the expected direction with a small effect size (Cohen’s *d* = 0·2).

**Table 3:** Strong Families Start at Home: baseline primary and secondary outcomes, overall and by intervention arm

Outcome	Maximum points	Overall (*n* 63)		Control group (*n* 30)		Intervention group (*n* 33)		*P*-value
		Mean	sd	Mean	sd	Mean	sd	
Primary outcome: child diet quality								
Total score Healthy Eating Index†	100	61·06	12·04	63·16	10·23	59·15	13·34	0·20
Healthy Eating Index 2015 components*								
Total fruits	5	3·88	1·61	3·93	1·60	3·82	1·64	0·79
Whole fruits	5	3·42	1·91	3·41	1·97	3·43	1·88	0·97
Total vegetables	5	1·77	1·45	1·86	1·31	1·69	1·58	0·66
Greens and beans	5	2·37	2·16	2·73	2·15	2·04	2·15	0·21
Whole grains	10	3·89	3·54	4·42	3·96	3·42	3·10	0·28
Dairy	10	8·29	2·73	8·18	2·59	8·40	2·89	0·76
Total protein foods	5	3·94	1·65	3·89	1·63	4·00	1·70	0·80
Seafood and plant protein	5	2·40	2·12	2·70	2·18	2·12	2·06	0·29
Fatty acids	10	3·59	3·29	4·18	3·31	3·05	3·24	0·18
Sodium	10	6·85	3·01	6·71	3·24	6·98	2·83	0·73
Refined grains	10	6·49	3·21	6·53	2·99	6·45	3·44	0·92
Added sugars	10	7·93	2·35	7·79	2·16	8·05	2·55	0·66
Saturated fats	10	6·23	3·13	6·82	2·95	5·69	3·24	0·16
Secondary outcome: parental feeding practices								
Food parenting inventory (FPI)‡								
Encourage to try new foods	5	3·47	0·84	3·47	0·67	3·47	0·98	0·97
Encourage exploration of new foods	5	3·32	1·13	3·21	1·26	3·42	1·01	0·46
Urge child to eat new foods	5	3·71	0·88	3·56	1·00	3·85	0·75	0·20
Repeated presentation of new foods	5	3·40	1·07	3·34	1·02	3·45	1·12	0·69
Family meals	5	3·94	0·87	3·88	0·93	4·00	0·83	0·60
Regular timing of meals and snacks	5	3·72	1·04	3·79	0·88	3·67	1·17	0·64
Inconsistent mealtimes	5	2·33	0·83	2·38	0·83	2·28	0·85	0·65
Indifferent feeding	5	2·74	1·16	2·83	1·15	2·66	1·17	0·57
Child involvement in food preparation	5	1·89	0·92	2·02	1·02	1·77	0·82	0·28
Pressure to eat	5	3·68	1·26	3·68	1·32	3·69	1·22	0·98
Restriction	5	4·16	1·02	4·24	0·93	4·08	1·12	0·53
Food as a reward	5	3·02	1·12	2·96	1·15	3·07	1·10	0·69
Responsiveness to child’s fullness cues	5	3·74	1·17	3·50	1·22	3·95	1·09	0·13
Monitoring	5	3·41	1·17	3·49	1·17	3·34	1·19	0·63
Comprehensive feeding practices questionnaire (CFPQ)‡								
Healthy eating guidance	5	4·05	0·73	4·01	0·76	4·09	0·71	0·69
Distraction	5	2·40	1·07	2·31	1·09	2·49	1·05	0·51
Parent socio-emotional context of feeding questionnaire‡								
Coercive	5	0·93	0·81	0·84	0·73	1·01	0·88	0·41
Support	5	2·38	0·96	2·34	0·93	2·42	1·00	0·74
Chaos	5	1·24	0·83	1·29	0·82	1·19	0·85	0·65
Structure	5	3·24	0·73	3·34	0·65	3·15	0·80	0·31

* Higher scores indicate higher diet quality.

† Higher scores indicate higher diet quality.

‡ Items are rated on a five-point Likert scale ranging from 1 (*never*) to 5 (*always*) and 1 (*disagree*) to 5 (*agree*). Higher subscale scores indicate greater use of that food parenting practice.

**Table 4:** Strong Families Start at Home: between-group differences of study outcomes at 6-month follow-up adjusted for baseline values

Outcome	PE	se	Effect size (Cohen’s *δ*)	95 % CI
Primary outcome: child diet quality				
Total score Eating Index	−0·55	4·69	−0·04	−0·67, 0·59
Healthy Eating Index 2015 components				
Total fruits	1·71	0·67	0·82	0·16, 1·47*
Whole fruits	2·14	0·83	0·83	0·17, 1·48*
Total vegetables	−0·35	0·49	−0·23	−0·86, 0·41
Greens and beans	0·06	0·88	0·02	−0·61, 0·65
Whole grains	0·83	1·1	0·25	−0·39, 0·88
Dairy	−0·90	0·85	−0·34	−0·97, 0·30
Total protein foods	0·22	0·42	0·17	−0·46, 0·80
Seafood and plant protein	−0·12	0·85	−0·04	−0·67, 0·59
Fatty acids	−0·31	1·12	−0·09	−0·72, 0·54
Sodium	−2·09	0·97	−0·70	−1·35, −0·04*
Refined grains	−0·80	1·16	−0·22	−0·85, 0·42
Added sugars	−0·28	1·13	−0·08	−0·71, 0·55
Saturated fats	−0·52	0·86	−0·20	−0·83, 0·44
Secondary outcome: food parenting practices				
Food parenting inventory (FPI)				
Encourage to try new foods	−0·09	0·20	−0·15	−0·78, 0·48
Encourage exploration of new foods	−0·09	0·31	−0·09	−0·72, 0·54
Urge child to eat new foods	−0·25	0·29	−0·28	−0·91, 0·36
Repeated presentation of new foods	−0·27	0·24	−0·36	−1·00, 0·28
Family meals	0·09	0·19	0·16	−0·47, 0·79
Regular timing of meals and snacks	1·08	0·27	1·31	0·61, 2·00*
Inconsistent mealtimes	0·03	0·29	0·03	−0·60, 0·66
Indifferent feeding	0·21	0·24	0·28	−0·36, 0·91
Child involvement in food preparation	−0·03	0·24	−0·04	−0·67, 0·59
Pressure to eat	−0·36	0·24	−0·49	−1·13, 0·16
Restriction	−0·35	0·31	−0·37	−1·01, 0·27
Food as a reward	−0·54	0·25	−0·70	−1·35, −0·04*
Responsiveness to child’s fullness cues	0·24	0·34	0·23	−0·41, 0·86
Monitoring	0·14	0·41	0·10	−0·53, 0·73
Comprehensive feeding practices questionnaire (CFPQ)				
Healthy eating guidance	0·06	0·24	0·09	−0·54, 0·72
Distraction	−0·79	0·29	−0·86	−1·52, −0·19*
Parent socio-emotional context of feeding questionnaire				
Coercive	0·28	0·18	0·49	−0·16, −1·13
Support	0·73	0·28	0·85	0·18, 1·51*
Chaos	0·21	0·24	0·28	−0·36, 0·91
Structure	0·25	0·20	0·40	−0·24, 1·04

PE, point estimate; se, standard error.

* Significant *P* value <0·05.

Effect size cut-offs: .20= small, .50 = moderate, .80 = large. Cohen, J. (1988). Statistical power analysis for the behavioral sciences (2nd ed.). Hillsdale, NJ: Lawrence Earlbaum Associates.

### Secondary outcome: food parenting practices

Treatment effects for secondary outcomes are also shown in Table 4. Effect sizes were significantly different from zero in food parenting practices for regular timing of meals and snacks (PE = 1·08, se = 0·27,95 % CI (0·61, 2·00)) that survived multiplicity correction, as well as nominally significant effects on reducing distractions during mealtimes (PE = -0·79, se = 0·29, 95 % CI (−1·52, −0·19)), less use of food as a reward (PE = -0·54, se = 0·25, 95 % CI (−1·35, −0·04)) and providing an autonomy-supportive feeding environment (PE = 0·73, se = 0·28, 95 % CI (0·18, 1·51)) (Table 4). Other factors that changed in the expected direction with medium effect sizes (Cohen’s *d* = 0·5) included using less pressure and those with small effect sizes (Cohen’s *d* = 0·2) included having family meals, using less restriction, using more responsiveness to child cues, monitoring and providing healthy eating guidance.

## Discussion

The goal of this pilot study was to assess the feasibility, acceptability and preliminary efficacy of the home-based responsive food parenting practices/nutrition intervention, *Strong Families Start at Home/Familias Fuertes Comienzan en Casa*. Despite COVID-19 interruptions, we found the intervention to be feasible and acceptable. In addition, we found preliminary positive effects on the child’s whole and total fruit consumption and some supportive food parenting practices. These preliminary outcome results can be used to inform the planning and possible modifications of a future fully powered randomised controlled trial.

We planned to deem our study feasible if we were able to recruit 100 % of the participants and retain 80 % of the participants at 6 months. We were successful with our recruitment targets, but the extreme disruption of the COVID-19 pandemic impacted our study mid-way. While we were able to adapt quickly and continue the study, we encountered greater difficulty reaching participants who either did not return our calls or stated they were too overwhelmed to continue with the study due to lack of childcare and/or homeschooling their children. Other studies have demonstrated that the pandemic impacted the desire to participate in health-related intervention research and increased difficulty to adhere to behavioural recommendations^(28)^. In our study, 34 % of our participants were from Pawtucket and Central Falls, RI, two of the cities hardest hit with COVID-19 in the USA^(29)^. Under these circumstances, we considered that retaining 63 % of participants from baseline to follow-up was acceptable. Importantly, we were more likely to retain Spanish-speaking participants, those born outside the USA, and those at risk for poor diet quality in terms of fatty acid and saturated fat components. Retention of such groups is uncommon in randomised trials and may reflect our recruitment and intervention strategies which included using bilingual, bicultural staff, working closely with community-based organisations from our community advisory board, using culturally relevant materials, and the important role of the CHW in connecting with these participants. Others have also found that CHW help to retain Hispanic/Latinx participants in research^(30)^.

The intervention was delivered with high fidelity across study design, training, intervention delivery, intervention receipt and enactment. Regarding intervention delivery, for example, almost all CHW used MI appropriately, completed the goal sheets, reviewed educational materials, built rapport with participants and completed the activities. CHW also reported high rates of participant engagement, although for phone calls this was lower. For intervention receipt and enactment, participants reported high rates of reading handouts and text messages. These findings are similar to other intervention studies that have utilised CHW to deliver the intervention^(31–33)^. For example, ANDALE, a home-based obesity prevention intervention, found that CHW met high levels of fidelity for providing a positive environment and feedback^(32)^. Similarly, the Entre Familia Reflejos de Salud study also found that the promotora-delivered intervention was delivered with high fidelity, including dose delivered and dose received^(33)^. In discussions with our CHW, phone calls were often challenging given that parents were often engaged in other activities and hence engagement was lower. Future studies should continue to understand the best modalities to engage with parents who have many competing demands.

The intervention was also highly acceptable; participants reported that the text messages and materials were very useful. Those who completed the final assessments were highly satisfied with the intervention overall as well as with the individual components. These findings are similar to other home-based studies wherein participants reported high levels of satisfaction with a home-based approach^(34,35)^. CHW have a long history of reaching traditionally under-resourced communities and have shown to be key components of many behavioural interventions with Hispanic/Latinx as they share the community’s background and language and understand the needs of the community^(18)^.

The overall diet quality of children in our sample was 61·6 which is equivalent to a grade D (less than satisfactory performance) and consistent with the national average for 2–4 year old children^(36)^. Although this pilot study was not powered to assess significant primary outcome changes, we did find meaningful changes with large effect sizes in total fruit and whole fruit consumption in the expected direction. Given that many US children are not meeting recommendations for fruit, especially children from low-income households, this change is important^(1)^. Furthermore, the COVID-19 pandemic has further exacerbated changes in diet quality and eating habits among families, especially among those that are low-income and ethnically diverse^(37)^. These results are somewhat similar to prior intervention studies^(32,35,38)^, which suggest that fruit consumption in children is easier to improve as compared with other dietary components^(35,39)^. Unlike other interventions targeting changes in food parenting practices and diet in young children, effect sizes in vegetable consumption were small^(32,33)^. These differential findings may be explained by the variation in measurement of child’s dietary intake across studies, with several interventions relying on self-reported questionnaires rather than gold standard 24-h recalls. Future studies might consider using biomarkers like dermal carotenoids to capture usual F&V intake.

We observed changes in the sodium component in an unanticipated direction. A post hoc analysis revealed that the decrease in the mean HEI-2015 sodium component score (indicating an increase in sodium intake) for those in the intervention group was attributed to intake of foods cooked at home with added salt (rice and chicken), tomato sauce and fast food. Although there were changes in this component favouring the control group, children from both groups were still compliant within the Dietary Guidelines for Americans 2015–2020 recommendations for their age group of 1500–1900 mg/d. The food parenting/nutrition intervention did include education on limiting consumption of fast food and salty snack foods (chips, etc.), but did not focus on decreasing the amount of salt used in cooking and other high sodium food products (such as canned tomato sauce) in the home. This finding suggests that future interventions in this population might consider including education on foods that are contributing to sodium intake which may be particularly important for Hispanic/Latinx populations where average sodium intakes exceed recommendations^(40)^. Given our null results in solid fats and added sugars, future iterations of this intervention should consider adding materials related to foods high in these nutrients.

Even though the study was not powered for statistical significance, we did find some changes to responsive food parenting practices including increased reports of structure (regular timing of meals and snacks). Previous studies have highlighted the importance of daily eating routines in facilitating the development of children’s competence to engage in healthy eating behaviours^(41,42)^. Routines are important for socio-emotional development in the preschool age group and can influence child behaviour at meals (e.g. if a snack is offered at a predictable time)^(4)^. Consistent mealtime routines are also associated with increased odds of preparing more meals at home^(43)^, which are of higher diet quality^(44)^. Other family-based nutrition interventions among older children (ages 5–8)^(45)^ have also reported similar results in setting rules and routines about healthy eating, and preparing food at home, and demonstrated a significant effect on children’s F&V intake.

We also found improvements in providing an autonomy-supportive feeding environment (involving children in food selection and preparation and encouraging children to express their food preferences and feelings of hunger and fullness). As children grow, a supportive environment increases autonomous motivation in the child to engage in and maintain behaviours such as decreasing sugar-sweetened beverage intake and increasing F&V consumption in adolescence^(46,47)^. Recent guidelines and other research^(4)^ highlight the importance of these practices in shaping healthy eating behaviours. Furthermore, a recent meta-analysis of fifty-eight childhood obesity prevention studies found that autonomy-supportive practices significantly mediated intervention efficacy on BMI^(48)^.

We also found a decreased use of distractions like screen time during mealtimes and the use of food as a reward. A systematic review of thirteen studies, including 3011 preschool-aged children, found that watching television during meal or snack consumption was associated with poorer diet quality (increased consumption of sugar-sweetened beverages, energy-dense nutrient-poor foods and fewer F&V) and weight status^(49)^. Using food as a reward for desired behaviour has been associated with decreased child development of food preferences from exposure alone, increased snack consumption and may predict an increase in BMI *Z* score over time^(21)^. Our findings are similar to that of the KAN-DO study a, mail-delivered intervention conducted with predominantly White, higher income families with preschoolers in that they also found a significant decrease in using food as rewards^(50)^. Other studies conducted with older Hispanic/Latinx children have found that increased parental monitoring, reinforcement and control strategies of their child’s diet were related to subsequent changes in young children’s consumption of F&V^(51)^. In the InFANT trial conducted with mothers and their infants up until 2 years of age, higher maternal feeding knowledge and lower use of food as a reward were found to mediate the direct intervention effect on child diet quality^(52)^. Similarly, the use of food as a reward was a significant mediator by which a telephone-based intervention influenced the consumption of unhealthy foods in the home with families of preschoolers in Australia^(53)^. While our study was not powered to assess mediation, a future fully powered trial should explore possible mediators to changes in children’s diet quality, such as parental diet, knowledge, outcome expectancies, autonomy support, relatedness, and competence.

In consideration of our study findings, there are some limitations worth noting. First, given the COVID-19 pandemic, several modifications had to be made to the intervention and data collection procedures. We were not able to test our fully home-based model and were unable to assess changes in the home food environment. Future research should be prepared to quickly pivot under emergency situations, including having clear protocols in place before the beginning of the intervention. Second, although HEI-2015 is widely used to assess diet quality in children and considers the correlation between each component and energy, there are some limitations that may have impacted its ability to accurately reflect change in diet quality in this current study. This pilot was not powered to detect statistically significant differences on component scores.

Although the 24-h recalls were conducted during a weekday and a weekend day to capture more variability in diet, these recalls may not be representative of a person’s usual diet, and they are prone to recall and social desirability bias. While advanced statistical methods have been developed to adjust for these limitations, and better reflect usual intake, they require a larger sample size than was available in this pilot study. Future studies could also consider adding objective measures of dietary intake, such as dermal carotenoid measurements, which can provide an objective measure reflecting fruit and vegetable intake. Third, the participants in this study were mostly from three cities in Rhode Island, limiting the generalisability of results to other geographic regions. Finally, it is important to note that almost half of the participants experienced food insecurity and most participated in food assistance programmes. The strategies discussed in the intervention assumed access to healthy foods, which may not always be the case. Future research should consider adding a food access component and/or connecting families to existing food safety net resources. In addition, it will be important to explore mediators, such as psychosocial variables, to understand the mechanisms for observed changes, as well as moderators of changes, such as acculturation, food security status and eating behaviour characteristics among others, to identify factors that might be related to a more positive intervention response in food parenting practices and ultimately children’s dietary quality.

This home-based food parenting/nutrition intervention was acceptable and feasible, given the challenges posed by the COVID-19 pandemic. We recruited and retained those at the highest risk for poor diet quality and saw significant changes in important primary and secondary outcomes. A larger fully powered future trial is needed with some modifications.

## Supplementary material

For supplementary material accompanying this paper visit https://doi.org/10.1017/S1368980023000174. click here to view supplementary material
