# Supplementary material for: Results from the Strong Families Start at Home/Familias Fuertes Comienzan en Casa: feasibility randomised control trial to improve the diet quality of low-income, predominantly Hispanic/Latinx children
Source: Public Health Nutr. 2023 Jan 24;26(4):890–904. doi: 10.1017/S1368980023000174 (PMC10131154; doi:10.1017/S1368980023000174)
Supplement: Supplementary file 1 [file S1368980023000174sup.zip › S1368980023000174sup001.docx]

Additional file 1. Strong Families Start at Home: Differences in baseline characteristics by retention status at 6 months.

|  | **n (%)** | | | **p-value** |
| --- | --- | --- | --- | --- |
| **Characteristic** | **Overall**  **(n=63)** | **Dropouts**  **(n=23)** | **Retained**  **(n=40)** |  |
| Baseline assessments completed |  |  |  | 0.044* |
| In Spanish | 39 (61.9) | 10 (43.5) | 29 (72.5) |  |
| Group |  |  |  | 0.18 |
| Healthy Eating | 33 (52.4) | 9 (39.1) | 24 (60.0) |  |
| Gender of target child |  |  |  |  |
| Female | 28 (44.4) | 10 (43.5) | 18 (45.0) | 1.00 |
| Gender of parent/guardian |  |  |  | 1.00 |
| Female | 58 (92.1) | 21 (91.3) | 37 (92.5) |  |
| Relationship to child |  |  |  | 1.00 |
| Mother | 57 (90.5) | 21 (91.3) | 36 (90.0) |  |
| Father or other | 6 (9.5) | 2 (8.7) | 4 (10.0) |  |
| Parent age, mean (SD) | 34.48 (7.6) | 34.04 (8.4) | 34.73 (7.2) | 0.74 |
| Ethnicity |  |  |  | 1.00 |
| Hispanic/Latino | 55 (87.3) | 20 (87.0) | 35 (87.5) |  |
| Not Hispanic/Latino | 8 (12.7) | 3 (13.0) | 5 (12.5) |  |
| Race |  |  |  | 0.65 |
| White | 24 (38.1) | 8 (34.8) | 16 (40.0) |  |
| Multiracial | 11 (17.5) | 5 (21.7) | 6 (15.0) |  |
| Unknown | 15 (23.8) | 4 (17.4) | 11 (27.5) |  |
| Other ^a^ | 13 (20.6) | 6 (26.1) | 7 (17.5) |  |
| Country of birth |  |  |  | 0.01* |
| United States | 24 (38.1) | 14 (60.9) | 10 (25.0) |  |
| Other | 39 (61.9) | 9 (39.1) | 30 (75.0) |  |
| Language used at home |  |  |  | 0.048* |
| English | 21 (33.3) | 12 (52.2) | 9 (22.5) |  |
| Spanish | 41 (65.1) | 11 (47.8) | 30 (75.0) |  |
| Missing | 1 (1.6) | 0 (0.0) | 1 (2.5) |  |
| Annual Household Income |  |  |  | 0.57 |
| Less than $25,000 | 34 (54.0) | 15 (65.2) | 19 (47.5) |  |
| Between $25,000 – 74,999 | 20 (31.7) | 5 (21.7) | 15 (37.5) |  |
| More than $75,000 | 3 (4.8) | 1 (4.3) | 2 (5.0) |  |
| Unknown | 6 (9.5) | 2 (8.7) | 4 (10.0) |  |
| Highest level of education |  |  |  | 0.043* |
| Less than 8th grade | 9 (14.3) | 2 (8.7) | 7 (17.5) |  |
| High School ^b^ | 23 (36.5) | 13 (56.5) | 10 (25.0) |  |
| College ^c^ | 31 (49.2) | 8 (34.8) | 23 (57.5) |  |
| Employment status |  |  |  | 0.64 |
| Employment full time | 15 (23.8) | 7 (30.4) | 8 (20.0) |  |
| Employment part time | 13 (20.6) | 4 (17.4) | 9 (22.5) |  |
| Other ^d^ | 35 (55.6) | 12 (52.2) | 23 (57.5) |  |
| Marital status |  |  |  | 0.21 |
| Married | 28 (44.4) | 7 (30.4) | 21 (52.5) |  |
| Not married ^e^ | 25 (39.7) | 12 (52.2) | 13 (32.5) |  |
| Missing | 10 (15.9) | 4 (17.4) | 6 (15.0) |  |
| Currently living with a spouse/partner |  |  |  | 0.26 |
| Yes | 43 (68.3) | 13 (56.5) | 30 (75.0) |  |
| No | 17 (27.0) | 9 (39.1) | 8 (20.0) |  |
| Missing | 3 (4.8) | 1 (4.3) | 2 (5.0) |  |
| Number of other adults living in home |  |  |  | 0.19 |
| None | 5 (7.9) | 3 (13.0) | 2 (5.0) |  |
| One | 15 (23.8) | 7 (30.4) | 8 (20.0) |  |
| Two or more | 42 (66.7) | 12 (52.2) | 30 (75.0) |  |
| Missing | 1 (1.6) | 1 (4.3) | 0 (0.0) |  |
| Number of children (<18 y) living at home |  |  |  | 0.41 |
| One | 18 (30.2) | 5 (21.7) | 14 (35.0) |  |
| Two or more | 44 (69.8) | 18 (78.3) | 26 (65.0) |  |
| Currently pregnant |  |  |  | 0.46 |
| Yes | 7 (11.1) | 2 (8.7) | 5 (12.5) |  |
| No | 49 (77.8) | 17 (73.9) | 32 (80.0) |  |
| Missing | 7 (11.1) | 4 (17.4) | 3 (7.5) |  |
| Food Assistance |  |  |  | 0.08 |
| Yes ^f^ | 51 (81.0) | 22 (95.7) | 29 (72.5) |  |
| No | 11 (17.5) | 1 (4.3) | 10 (25.0) |  |
| Missing | 1 (1.6) | 0 (0.0) | 1 (2.5) |  |
| Food insecurity |  |  |  | 0.88 |
| Yes ^g^ | 28 (44.4) | 11 (47.8) | 17 (42.5) |  |
| Target child attends childcare |  |  |  | 0.07 |
| Yes | 20 (31.7) | 11 (47.8) | 9 (22.5) |  |

^a^ Includes: Black/African American, American Indian/Alaskan Native, Asian, Hawaiian/Other Pacific Islander, other.

^b^ Includes some high school, high school graduate or General Educational Diploma (GED), post high school trade or technical school.

^c^ Includes some college and college graduate or higher.

^d^ Other includes employed seasonally, unemployed/looking for work, student, homemaker and disable.

^e^ Not married includes never married, separated, divorced, widowed.

^f^ Includes Supplemental Nutrition Assistance Program (SNAP), Supplemental Nutrition Program for Women and Infants (WIC), free/reduced price school meal, soup kitchen, food pantry.

^g^ Includes any level of food insecurity (“We worried whether our food would run out before we got money to buy more; the food we bought just didn't last, and we didn't have money to get more.”)

*Significant p-value <0.05; **Significant p-value <0.01; ***Significant p-value <0.001
