# Supplementary material for: Results from the Strong Families Start at Home/Familias Fuertes Comienzan en Casa: feasibility randomised control trial to improve the diet quality of low-income, predominantly Hispanic/Latinx children
Source: Public Health Nutr. 2023 Jan 24;26(4):890–904. doi: 10.1017/S1368980023000174 (PMC10131154; doi:10.1017/S1368980023000174)
Supplement: Supplementary file 1 [file S1368980023000174sup.zip › S1368980023000174sup003.docx]

Additional file 2. Strong Families Start at Home: Differences in baseline values of primary and secondary outcomes by retention status at 6 months.

|  | **Maximum**  **points** | **Mean (SD)** | | | **p-value** |
| --- | --- | --- | --- | --- | --- |
| **Outcome** |  | **Overall**  **(n=63)** | **Dropouts**  **(n=23)** | **Retained (n=40)** |  |
| **Primary outcome: Child Diet Quality** | |  |  |  |  |
| *Healthy Eating Index 2015 components* ^a^ | | |  |  |  |
| Total Fruits | 5 | 3.88 (1.61) | 3.96 (1.56) | 3.83 (1.66) | 0.76 |
| Whole Fruits | 5 | 3.42 (1.91) | 3.28 (1.93) | 3.50 (1.91) | 0.67 |
| Total Vegetables | 5 | 1.77 (1.45) | 1.63 (1.34) | 1.86 (1.52) | 0.54 |
| Greens and Beans | 5 | 2.37 (2.16) | 2.65 (2.26) | 2.20 (2.12) | 0.44 |
| Whole Grains | 10 | 3.89 (3.54) | 3.55 (3.78) | 4.10 (3.43) | 0.56 |
| Dairy | 10 | 8.29 (2.73) | 8.56 (2.70) | 8.13 (2.78) | 0.55 |
| Total Protein Foods | 5 | 3.94 (1.65) | 3.93 (1.49) | 3.95 (1.77) | 0.97 |
| Seafood and Plant Protein | 5 | 2.40 (2.12) | 2.52 (2.27) | 2.32 (2.04) | 0.74 |
| Fatty Acids | 10 | 3.59 (3.29) | 2.16 (2.55) | 4.46 (3.41) | 0.007* |
| Sodium | 10 | 6.85 (3.01) | 6.71 (3.34) | 6.95 (2.84) | 0.77 |
| Refined Grains | 10 | 6.49 (3.21) | 6.19 (3.01) | 6.67 (3.35) | 0.58 |
| Added Sugars | 10 | 7.93 (2.35) | 7.81 (2.02) | 8.00 (2.56) | 0.76 |
| Saturated Fats | 10 | 6.23 (3.13) | 4.44 (3.13) | 7.31 (2.63) | <0.001* |
| *Total Score Healthy Eating Index* ^b^ | 100 | 61.06 (12.04) | 57.39(11.15) | 63.28(12.15) | 0.06 |
| *Calories (kcal)* |  | 2036 (890) | 2040 (964) | 2034 (855) | 0.98 |
| **Secondary outcome: Parental Feeding Practices** | | | |  |  |
| *Food Parenting Inventory (FPI)* ^c^ | |  |  |  |  |
| Encourage try new foods | 5 | 3.47 (0.84) | 3.28 (0.78) | 3.58 (0.86) | 0.16 |
| Encourage exploration of new foods | 5 | 3.32 (1.13) | 3.17 (1.17) | 3.41 (1.12) | 0.43 |
| Urge child to eat new foods | 5 | 3.71 (0.88) | 3.72 (0.95) | 3.71 (0.85) | 0.98 |
| Repeated presentation of new foods | 5 | 3.40 (1.07) | 3.32 (0.91) | 3.45 (1.15) | 0.64 |
| Family meals | 5 | 3.94 (0.87) | 3.93 (0.95) | 3.95 (0.84) | 0.95 |
| Regular timing of meals and snacks | 5 | 3.72 (1.04) | 3.83 (0.96) | 3.67 (1.09) | 0.56 |
| Inconsistent mealtimes | 5 | 2.33 (0.83) | 2.22 (0.73) | 2.39 (0.89) | 0.43 |
| Indifferent feeding | 5 | 2.74 (1.16) | 2.41 (0.86) | 2.92 (1.27) | 0.10 |
| Child involvement in food preparation | 5 | 1.89 (0.92) | 1.62 (0.82) | 2.04 (0.95) | 0.08* |
| Pressure to eat | 5 | 3.68 (1.26) | 3.81 (1.14) | 3.62 (1.33) | 0.57 |
| Restriction | 5 | 4.16 (1.02) | 4.10 (0.97) | 4.19 (1.07) | 0.74 |
| Food as a reward | 5 | 3.02 (1.12) | 2.84 (1.08) | 3.12 (1.14) | 0.35 |
| Responsiveness to child's fullness cues | 5 | 3.74 (1.17) | 3.77 (1.26) | 3.72 (1.13) | 0.87 |
| Monitoring | 5 | 3.41 (1.17) | 3.32 (1.27) | 3.47 (1.13) | 0.63 |
| *Comprehensive Feeding Practices Questionnaire (CFPQ)* ^c^ | | | | | |
| Healthy eating guidance | 5 | 4.05 (0.73) | 3.92 (0.69) | 4.12 (0.75) | 0.31 |
| Distraction | 5 | 2.40 (1.07) | 2.45 (1.14) | 2.37 (1.04) | 0.80 |
| *Parent Socioemotional Context of Feeding Questionnaire* ^c^ | | | | | |
| Coercive | 5 | 0.93 (0.81) | 0.83 (0.77) | 0.98 (0.83) | 0.90 |
| Support | 5 | 2.38 (0.96) | 2.24 (1.03) | 2.46 (0.92) | 0.38 |
| Chaos | 5 | 1.24 (0.83) | 1.34 (0.81) | 1.18 (0.85) | 0.47 |
| Structure | 5 | 3.24 (0.73) | 3.31 (0.60) | 3.20 (0.81) | 0.56 |

^a^ Higher scores indicate higher diet quality.

^b^ Higher scores indicate higher diet quality.

^c^ Items are rated on a 5-point Likert scale ranging from 1 (never) to 5 (always) and 1 (disagree) to 5 (agree). Higher subscale scores indicate greater use of that child feeding practice.

*Significant p value <0.10
